# Incorporating management opinion in green supplier selection model using quality function deployment and interactive fuzzy programming

**DOI:** 10.1371/journal.pone.0268552

**Published:** 2022-06-16

**Authors:** Beenish Khan Khattak, Afshan Naseem, Mehran Ullah, Muhammad Imran, Sami El Ferik

**Affiliations:** 1 Department of Engineering Management, College of Electrical and Mechanical Engineering (CEME), National University of Sciences and Technology (NUST), Islamabad, Pakistan; 2 Interdisciplinary Research Center for Smart Mobility and Logistics, King Fahd University of Petroleum & Minerals, Dhahran, Saudi Arabia; 3 Department of Operations and Supply Chain, NUST Business School, National University of Sciences and Technology (NUST), Islamabad, Pakistan; 4 Control & Instrumentation Engineering Department, King Fahd University of Petroleum & Minerals, Dhahran, Saudi Arabia; Sunway University, MALAYSIA

## Abstract

The need for environmental protection and involvement of ecological aspects in the business operations is forcing the organizations to re-examine their action plans and rebuild their supply chain activities. Many organizations are incorporating environmental rules and regulations in their everyday matters by focusing on green supplier selection. The proposed research paper develops a multi-objective interactive fuzzy programming model for the selection of suppliers. This model works on a business quartet of green appraisal score, cost, quality, and time. The model uses an environmental scale for different green parameters and all the suppliers are scored based on this scale. In this research model, Quality Function Deployment (QFD) methodology is integrated with the multi-objective interactive fuzzy programming. QFD technique is utilized to compute the weights of several green factors used for the selection of suppliers. The model uses a Fuzzy linguistic scale and a triangular membership function to link expert opinions along with their experience to solve the problem. Finally, the model is validated on a numerical case study of the textile industry for green supplier selection which achieves a 100% satisfaction for cost and time, 75% satisfaction for green appraisal score, and 93.95% for the quality. The proposed model assists the decision-makers in selecting green suppliers to improve the overall sustainability of their organizations.

## 1. Introduction

Supply Chain Management (SCM) is a mixture of different activities starting from the utilization of raw materials to the delivery of end products in an organized manner. Organizations have started to evaluate their supply chains in response to various interlinked economic and environmental challenges [1, 2]. In the present world, an organization’s ecological efficiency is interlinked with its vendor’s ecological efficiency, and finding green suppliers is an important aspect in having a competitive edge over other organizations [3]. In an industrial context, the word “green” implies something that is not harmful to the environment and is synonymous with sustainability [4]. According to [5], sustainability can be considered as a capability of an organization to solve real-time problems and make decisions without any harmful impacts on the environment. Sustainability has gained a lot of attention in recent years [6, 7].

In today’s world, environmental factors are rapidly emerging as important parameters for the business community. Worldwide environmental policies exert significant pressure on the industries as a result of which organizations respond to these regulations by introducing items/services which utilize less or reused bundling, diminish contamination, or potentially less energy consumption [8]. Many firms are attempting to ensure that the operations and performances in their production houses; as well as those managed by their partners in the supply chain are more sustainable [9]. Firms around the world are adopting strategies such as “clean production” and “eco-efficiency” to respond to sustainability and its related issues [10]. One of the ways to reduce environmental deterioration is by linking ecological issues to the company’s purchasing decisions. The environmental deterioration also results sometimes in the product deterioration. The phenomena of deterioration are related to spoilage, damage, vaporization or other changes in product quality or productivity because of environmental changes during storage [11]. Integrating supply chain operations with environmental policies can be a suitable technique for improving the environmental performance of an industry. For this aspect, the idea of GSCM can be used here. GSCM is one of the important approaches to prevent the environment and was first introduced by [12]. GSCM is formed by integrating economy with ecology and protects the environment and its resources [13, 14]. As the organizations have started integrating their competitive objectives with the environmental policies, GSCM has been introduced in the latest research [15–20].

As stated by [21], the cost of raw materials constitutes a large amount in the total cost of the product, therefore the selection of suppliers has a direct influence on organizational efficiency, increasing profitability and cost reduction in various products. The cost of raw materials can be up to 70% of the total cost of the product in some of the organizations [22], therefore suppliers are an important pillar for the success of an organization. In today’s world, organizations have to focus on the environment along with the economic aspects because consumers desire not only to meet their expectations and needs but also demand respect for the environment [23]. Therefore, within the supplier selection process, it is recommended to select those suppliers that struggle to save the environment and focus on sustainability-related issues [24]. According to [25], the selection of green suppliers can help enterprises to prosper in terms of competitive advantage along with the traditional economic benefits. Sustainable supplier selection also helps in increasing product quality, customer satisfaction, and helps in the selection of strategic partnerships. While studying the relation between green supply chains and pricing strategies of a product, [26] claimed that environmental protection of a product is related to the environmental awareness of consumers; and the stronger the environmental consciousness of consumers, the higher the level of green degree will be ensured in a supply chain.

The supplier selection is performed based on specific criteria which are decided by the company stakeholders or experts from the management. The traditional criteria of supplier selection included factors like cost, quality, performance history of suppliers, service level, and lead time. [27] was the pioneer who recognized 23 distinct standards of supplier evaluation where the most significant were quality, conveyance, execution history, guarantees, or claims. As in recent years, the global ecological efficiency of organizations has become vital by considering the eco-friendly regulations along with the corporate goals, the integration of environmental and economic strategies is necessary. The selection of green suppliers can reduce the generation of pollutants from the source, and mitigate harmful climate changes [28]. Some of the green measures for supplier evaluation are regularly identified with the degree of wastewater release, carbon dioxide emissions, degree of reuse of solid waste, and degree of use of unsafe material [29]. [30] provided criteria for sustainable supply chain operations and barriers that need to be addressed while achieving these sustainable objectives.

Presently, the worldwide environmental efficiency of associations has gotten fundamental by considering the eco-friendly regulations along with the corporate goals, the integration of environmental and economic strategies is necessary [31]. This paper describes a procurement problem considering the green score ranking of suppliers along with cost, quality, and time. The model optimizes four objective functions: (1) minimizing the cost objective comprising of product, labor, energy, transportation, and carbon emission costs during logistics and handling of the product; (2) maximizing the quality objective comprising of the number of complaints received by each supplier for the last one year per million units of product sold; (3) minimizing the time objective comprising of the production, transportation and the quality inspection time; (4) maximizing the green appraisal scores comprising of various green parameters serving as the green criteria of supplier selection. These parameters of the green appraisal scores are industry-specific to some extent.

An environmental scale is also used for scoring green parameters through interviews with various experts in the industry. On the environmental scale, 1 refers to the supplier with bad environmental practices whereas 4 refers to the green supplier. The environmental parameters link to the three main green factors i.e. 1) Green design; 2) Green logistics and 3) Environmental Management System (EMS). QFD methodology is used to create House of Quality(s) (HOQs): first HOQ links the stakeholders with their requirements whereas second HOQ is used to relate the stakeholders’ requirements with green factors. QFD is used to calculate the weights of the green factors and the suppliers are ranked by the weighted fuzzy goal programming technique. This paper attempts to select and rank different suppliers for the textile industry in Pakistan. This paper uses a case study of the textile industry to apply the mathematical model and verify the results. The proposed hybrid model would be practically helpful to all industries in the supplier selection domain.

The rest of the proposed paper is composed as follows. A literature survey identified with the supplier selection problem incorporating the environmental criteria is described in Section 2. The characteristics of the proposed model are described in Section 3 whereas Section 4 shows the numerical experimentation and analyses of the proposed model. The conclusion and prospects are described in Section 5.

## 2. Literature review

Three primary areas of research are highlighted in this paper. The first area of research studies the integration of environmental regulations with supply chain operations and the evolution of multi-objective decision-making methodologies for the selection of suppliers. The second research area focuses on the supplier selection criteria that include traditional and environmental perspectives. The third research area integrates the problem with order allocation. This paper uses all three above-mentioned research areas therefore they are briefly reviewed in this section.

Many countries around the world have started introducing various policies and incentives for manufacturers to make efforts to reduce environmental pollution and move towards sustainability [32]. The production, transportation, and purchasing activities of manufacturing firms have a great impact on the environment [33]. Therefore, manufacturers have acknowledged the importance of growing dependable connections among the purchasers and vendors. For many organizations, the selection of suppliers is considered a vital component of purchasing and is generally perceived as a pivotal administration obligation [34]. The supplier selection process generally has 3 phases: 1) Pre-selection, 2) Selection, and 3) Post-selection [35]. The first phase deals with the needs of organizations to set corporate goals for selection. In the second stage, there should be some pre-set robust selection criteria as this step chooses the most suitable suppliers from a list of available suppliers. In the last stage, once the suppliers are selected, the organization starts corporate collaborations with them for achieving a mutual objective. The second stage of the supplier selection process is an important one as it requires some MCDM methodologies for analyzing the suppliers. [36] reviewed different supplier selection criteria and methods for the first time from 1966 to 1991. In 2001, [37] also reviewed these methods and presented some novel methodologies for the supplier selection framework. From 2000 to 2008, this area was reviewed by [38]. [39] reviewed this area from 2008 to 2012 and presented 26 decision-making techniques which he divided into three major types: (1) Mathematical programming techniques, (2) MCDM techniques, and (3) Artificial intelligence techniques.

The supplier selection process can be related to single sourcing or multiple sourcing. Single sourcing refers to problems where only one supplier is selected, whereas in multi-sourcing, more than one supplier is selected for the problem and the quantity of the order is determined among all suppliers [20]. Therefore, order allocation is also considered an important decision-making process in supply chain management. [40] combined the linear programming with the Fuzzy Analytic Network Process (ANP) for optimal allocation of quantity among selected suppliers. [41] developed a model for order allocation using fuzzy AHP and TOPSIS. For the order allocation, [42] utilized a goal programming technique for optimization of a multi-objective problem considering the price, rejects, and lead-time.

The multiple sourcing problems are generally complex and involve MCDM, therefore many researchers have applied different optimization techniques to solve these problems. Some of these techniques are Analytic Hierarchy Process (AHP) [43–45]; Multi-Attribute Decision Making (MADM) [46, 47]; Best Worst Method (BWM) [48, 49]; Analytic Hierarchy Process (ANP) [50, 51]; Data Envelopment Analysis (DEA) [52]; the Technique of Order Preference Similarity to the Ideal Solution (TOPSIS) [53–55]; Decision-Making Trial and Evaluation Laboratory (DEMATEL) [56, 57]; entropy [58–60]; and other many more [61–63].

Many researchers have started using the concept of hybridization. This technique is beneficial when several approaches are combined to tackle complex supplier choice issues [64–66]. Some of the hybrid models are the integrated Data Envelopment Analysis-Artificial Intelligence (DEA-AI) and Data Envelopment Analysis-Artificial Neural Network (DEA-ANN) technique [67]. During the past years, a major research trend is seen in dealing with the uncertainties in the supplier selection problems. Including the fuzzy approach in the research of supplier selection [3, 13, 28] and integrating different MCDM techniques with the fuzzy set theory [68–70]. [71] presented the Hierarchy Fuzzy TOPSIS based model for supplier selection including 25 parameters of selection. [72] developed a model combining fuzzy DEMATEL, fuzzy AHP, and fuzzy Delphi to solve a vendor selection problem. [73] proposed an integrated approach consisting of fuzzy AHP and fuzzy multi-objective linear programming in which fuzzy AHP was used for estimating the weights of supplier evaluation criteria. [74] used a multi-objective linear programming model for evaluating suppliers and allocating order quantities considering multi periods, multi-products, multi-modal transportation, shortages, and discount conditions. [66] used a hybrid MCDM and multi-objective mathematical programming model for supplier selection integrating ANP with DEMATEL.

Due to the increasing environmental deterioration, GSCM has recently evolved in academic research as well as business organizations. Organizations have started to integrate their corporate goals with environmental regulations to deal with market pressures and social responsibility. To improve environmental performance, organizations tend to incorporate these strategies into their vendor selection process. To provide eco-friendly end products, it is vital to include the green criteria in the selection of vendors hence GSCM becomes a critical concept in the supplier selection domain. In order to increase competitive advantage, the organizations must adopt strategies that can lead them to achieve their economic and environmental goals simultaneously [75]. Some researchers have included GSCM in their models which solve complex vendor selection problems [65, 76–78]. Green supplier selection is a common topic of research [19, 41, 79, 80]. Carbon emissions play a vital role in GSCM therefore carbon footprint is an important parameter for this evaluation process [46]. Waste management is directly related to GSCM [81]. [82] claimed that “to cope up with the expanding market pressures and requests from different stakeholders and to consent to more demanding environmental regulations, organizations have begun to examine their supply chains to improve their overall sustainability profile.” Therefore, a large group of experts has started to incorporate environmental issues in the process of selecting suppliers for different industries.

Companies consider different parameters such as quality, cost, and delivery time to assess their suppliers [83, 84]. However, the ecological issues have forced businesses to think over sustainable and social issues. As of now, numerous associations are practicing environmental safety in their organizations because of their apprehension for sustainability [85]. Green supplier selection is based on a wide range of criteria. Some other criteria include energy consumption, materials, liquid, and solid residue, and innovation as the major ecological measures [86]. Contamination control, green processes, green products, and ecological and legislative administration as the principal standards of the green selection of vendors were added in the GSCM research by [87]. 12 environmental criteria that include the use of environment-friendly technology, the use of environment-friendly materials, green market share, partnership with green organizations, green research and development projects, and staff training were introduced by [88].

Some other environmental criteria specified by different researchers are but not limited to: pollution due to air emissions, level of wastewater, and solid waste discharge [28, 61, 89–93]; noise level [90], degree of harmful material utilization [28, 90]. [15] proposed a model for the selection of suppliers based on the Triple Bottom Line (TBL) method. [3] decided on five parameters for assessing green suppliers including quality, cost, conveyance, innovation capacity, and natural competency. A green supplier saves energy and resources and focuses on green packing and green designing in supplier selection operations [65]. The selection of green suppliers is more complex as compared to traditional supplier selection [94, 95] since it requires consideration of qualitative and conflicting environmental criteria [96]. A green supplier uses resources efficiently, reduces waste and extra material usage, and uses less energy [23]. This research study will add value to the literature by determining the novel criteria for green supplier selection. This paper defines novel criteria for the selection of suppliers incorporating both traditional and environmental parameters which can be easily used by all industries for their procurement processes.

In the existing literature, there are several methods used by researchers for the green supplier evaluation under vague and uncertain environments. Some of the common mathematical modeling techniques can be divided into mathematical programming (goal programming, Mixed Integer Linear Programming (MILP), linear programming, non-linear programming); qualitative techniques, methods that use artificial intelligence (grey system theory, fuzzy logic, neural networks, and genetic algorithm), and analytical methods like DEA, ANP, AHP, TOPSIS [97]. [97] solved the GSS problem by using the fuzzy VIKOR method for finding different weights of criteria. [98] proposed a hybrid method for the selection of suppliers and order allocation in the paper industry using fuzzy TOPSIS and fuzzy Multi-Objective Linear Programming. [99] integrated fuzzy DEMATEL and ANP to deal with a GSS problem. [17] integrated QFD with DEMATEL for developing a supplier selection model. [100] proposed a framework combining DEMATEL with the Taguchi Loss function for assessing the performance of suppliers and calculating the value of each supplier.

Similarly, [101] used the multi-attribute utility theory (MAUT) and multi-objective linear programming (MOLP) to solve a multiple sourcing problem. [48] used Best Worst Method (BWM) to find the weights of parameters and modified fuzzy TOPSIS to rank, and fuzzy MODM to allocate the ordered quantity among the suppliers. [102] combined QFD with Elimination and Choice Expressing Reality (ELECTRE) for supplier selection in the quality management system. [103] solved a GSS problem through a fuzzy approach based on TOPSIS and entropy model. [104] linked fuzzy AHP and fuzzy TOPSIS methods for evaluating a GSS problem. [65] combined BWM and the VIKOR method to deal with a GSS problem. [105] evaluated the potential green suppliers by integrating QFD with partitioned Bonferroni mean operator. [63] combined fuzzy BWM with VIKOR to select suitable sustainable suppliers. [106] used BWM with MULTIMOORA for supplier selection in mining equipment manufacturing. [30] integrated AHP with ELECTRE to identify the major criteria for sustainable supply chain operations. [107] proposed a green supplier evaluation model under the uncertain environment and used a hesitant fuzzy TOPSIS method for the model. [108] considered different supplier evaluation criteria and ambiguities in criteria values while solving a sustainable supplier selection problem using DEMATEL and Fuzzy VIKOR methods. [7] proposed a model for evaluating the impact of green credit rating and its implications in SCM. The preceding studies have demonstrated that GSCM has been a vital topic for discussion in both academic research and the business world. The literature review of GSCM under fuzzy environments shows that fuzzy set theory is an important approach to deal with vagueness in the selection of suppliers. Table 1 shows the examination of paper in hand and the existing GSCM papers that have proposed vendor selection methods.

**Table 1:** GSCM literature.

Research	Supplier Selection Method	Application Domain
[3]	Fuzzy AHP, Fuzzy TOPSIS, and MOLP	Automobile Manufacturing company
[17]	DEMATEL, QFD and COPRAS	Kalleh Dairy company in Iran
[50]	ANP-QFD and MOORA	Dairy company
[88]	Fuzzy TOPSIS	Logistics
[97]	Intuitionistic fuzzy VIKOR	Manufacturing company
[98]	Fuzzy TOPSIS and MOLP	Paper industry
[100]	DEMATEL and Taguchi Loss functions	Online Retailer company
[105]	QFD with partitioned Bonferroni mean operator	Transportation industry
[106]	BWM with MULTIMOORA	Mining Equipment Manufacturing
[108]	DEMATEL and Fuzzy VIKOR	Management firm
[111]	DEA and ANP	High-tech industry
[112]	Fuzzy TOPSIS	Brazilian Electronics company
[113]	AHP and Fuzzy TOPSIS	Green service Food Manufacturing company in Iran
Proposed Research	QFD and Fuzzy Interactive Multi-Objective Weighted Programming	Textile Industry

As appeared in the literature survey, weights of criteria have frequently been determined by AHP or ANP, both requiring tedious pairwise comparisons and hence it is difficult to get reliable outcomes. The above issue can be addressed by the use of QFD to calculate the criteria weights. Although the proposed model is multi-objective with four equally important objectives, their importance varies from business to business. The four objective functions of the proposed model are cost, quality, time, and green appraisal score. The most important objective in the proposed model is the green score which serves in the context of environmental regulations for industrial organizations. The ranking of suppliers can be done by the optimization of these four objective functions based on expert opinions. Generally, the expert opinion of all the expert committee members is given equal weightage, whereas their knowledge, skill, and experience vary from each other [109].

Thus, to prioritize the objectives, the model considers human expert opinion weights subjected to everyone’s managerial experience regarding the procurement process. After finalizing the weights, the weighted goal programming is used for the optimization of these objectives to obtain an optimal solution. The model considers a novel environmental scale of different parameters important to be considered for a sustainable supply chain and production activities. The suppliers are scored for these environmental parameters based on this scale after quantifying the expert opinions; with 1 being a weak green supplier whereas 4 being a strong green supplier. [110] used a similar environmental scale to incorporate a green supplier selection criterion for finding optimal suppliers by following two different multi-objective algorithms to get Pareto-optimal solutions. Therefore, [110] paved way for this research paper presenting a novel systematic framework of supplier selection by integrating QFD with the weighted goal programming which has not yet been found in the literature of green supplier selection. The model is applied and verified on the textile industry in Pakistan whereas it can be applied to any industry for the selection of its suppliers.

## 3. Proposed model

This section describes the problem statement, assumptions, notations, and mathematical modeling for the green supplier selection and allocation.

### 3.1 Problem statement

The proposed research is a multi-objective problem of green supplier selection for the textile industry. The study considers a set of suppliers and a Green Textile Small and Medium Enterprise (GTSME) which is the purchaser of the dyestuff product to be in its manufacturing operations. As the textile dyestuffs contain harmful carcinogenic materials that need to be treated before leaving directly into the environment, therefore, GTSME tends to focus on those suppliers who use environment-friendly raw materials and have green policies about their manufacturing processes. The quality is inspected by the GTSME in terms of the type and degree of environment-friendly raw materials used for their production.

The GTSME gives the demand of the product to the suppliers whose quality of the product is inspected, and then when the quality check is completed, these potential suppliers are evaluated based on a business quartet of cost, quality, time, and green appraisal score/rank. After the evaluation of these suppliers, the GTSME communicates with the selected supplier(s) to place the order of the dyestuff product. It is important to discuss here that this model is proposed for a textile company, however, the model remains valid for any industry where supplier selection is involved. The criteria objectives cost, quality, time and green appraisal score/rank are conflicting objectives which require a trade-off among them for solving the problem. However, the Green Appraisal score is the main concern in the problem as the GTSME focuses on the selection of green suppliers. The overall vendor selection problem is described in the following steps:

1. GTSME generates a demand for a specific dyestuff product to the network of suppliers who are present in their contact information. GTSME sends a tender notice to all suppliers.
2. The GTSME assures the quality of these suppliers depending on the test results of the products according to their standard, and the quality reports are sent to the suppliers.
3. The GTSMEs then evaluate the qualified supplier(s) from step 2, based on green appraisal score/rank, cost, quality complaints, and time. These complaints may include the harmful toxic materials used in the production of the product, damaged or expired products causing harmful diseases to the workers of the textile industry, etc. The green score of suppliers is calculated by considering various environmental parameters being incorporated in the manufacturing processes of suppliers.
4. Finally, the GTSME gives a demand to the selected supplier(s) after the complete evaluation process is completed.

Both the economic and environmental criteria are equally important for the selection of suppliers but GTSME being an eco-friendly industry considers the green selection to be more important to select those suppliers who give the least harm to the environment by producing non-toxic and non-hazardous products as a result of which the GTSME would be using the environment-friendly materials in their manufacturing processes.

### 3.2 Green supplier selection criteria

**Fig 1:**
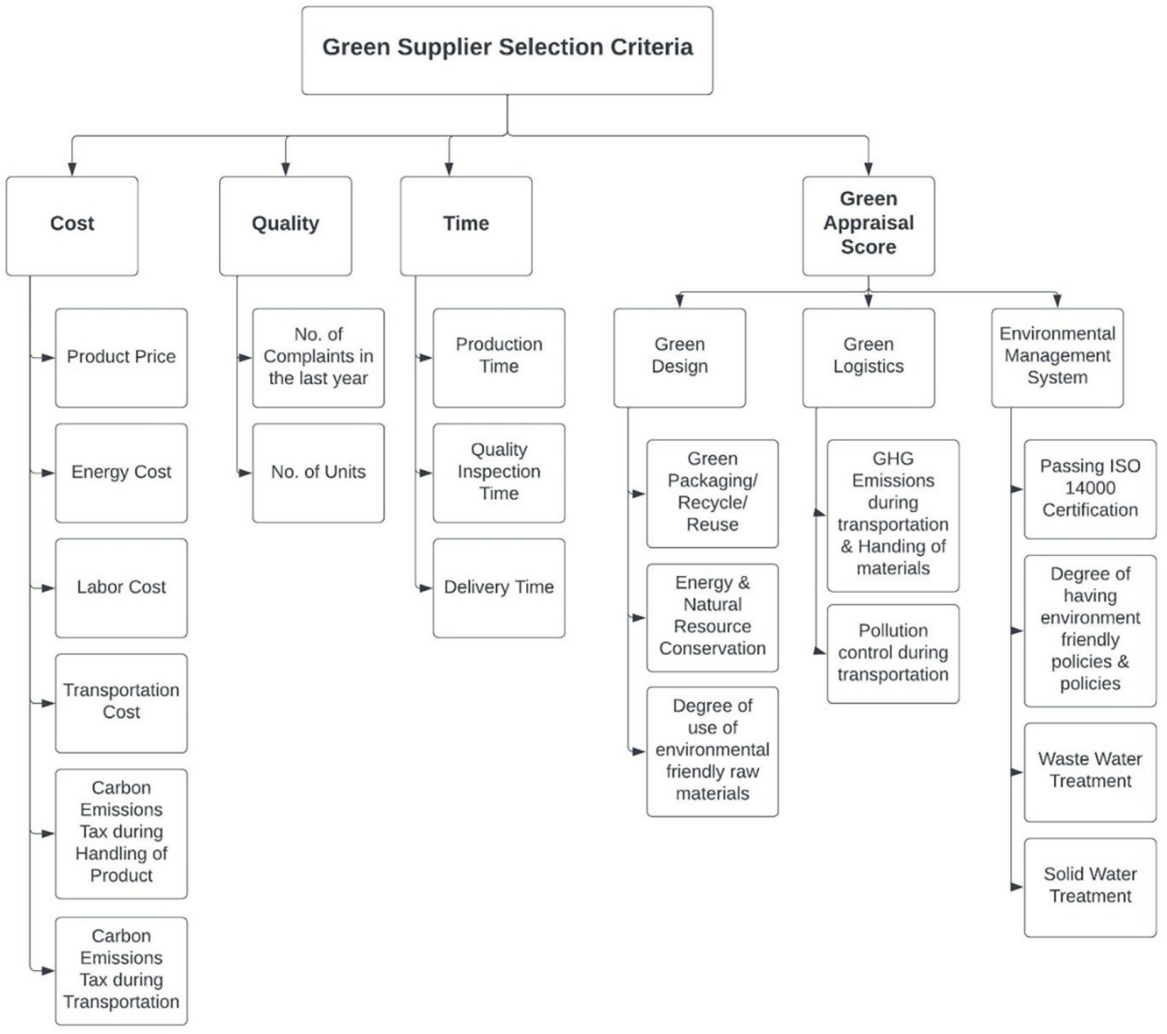
Green supplier selection criteria.

Various criteria of green supplier selection have been studied from the cited literature and discussed with the experts from the subject industry i.e., textile industry in this study. Specialists from the organization are consulted to accumulate data to distinguish the feasible factors and sub-factors. Fig 1 shows the important factors identified by expert managers from GTSMEs and verified from the literature, the proposed criteria is a *business quartet* which is divided into two types: a) environmental or green criteria; b) economic criteria including cost, quality, and time. The first and most important type of criterion on which suppliers are evaluated is the environmental and green criterion. This forms the basis of this research as environmental regulations have recently been a vital perspective to consider in industrial businesses. The green criterion is divided into three main green factors and then several parameters under these factors. The 3 main factors of green criteria are 1) Green Design (GD), 2) Green Logistics (GL), and 3) Environmental Management System (EMS). Furthermore, these factors include green or recycled packaging, degree of use of environmentally safe raw materials or chemicals, energy consumption, Carbon Dioxide (CO_2)_ emissions during manufacturing and transportation, and some environmental or eco-tech certifications like ISO 14000, and solid and wastewater treatments.

According to the experts of the industry, wastewater discharge and carbon emissions are the most important factors to be considered for this industry. A scale of 1 to 4 is formed for each parameter of green supplier selection criteria incorporating different states of each parameter given in Table A-1 in S1 Appendix. The scores of these parameters of green criteria add up to form a green appraisal score for the suppliers and all the suppliers are ranked based on this score along with three other economic objectives which are included in the second type of criterion. The cost of the supplier is evaluated by the price of products, transportation costs including the distance between the supplier and the purchaser, and the quality inspection cost. The quality of the supplier is evaluated by considering the number of complaints received by the supplier in the last year for a product. These quality complaints are uncertain and for this purpose, the fuzzy theory is applied to convert the uncertain quality complaints into crisp data. The time of the supplier is evaluated by calculating the sum of production, transportation, and quality inspection time. The weights of the criteria are decided after engaging in one-to-one interviews with industry experts. After this, a HOQ is applied to the criteria of supplier evaluation and the stakeholders’ requirements to find the weight of each parameter.

### 3.3 Model assumptions

The following are the important assumptions used to model the defined problem mathematically:

1. Demand is known and constant.
2. The price of the raw material and the capacity of the supplier remain fixed throughout the year.
3. The quality inspection cost is paid by the GTSME and remains constant.
4. The number of quality complaints received by the supplier for the last year is uncertain, thus taken as a triangular fuzzy number.
5. The number of units of the product sold in the last year is known

### 3.4 Model formulation

#### 3.4.1 Indices

**Table:** 

*g*	index for GTSME	*g* = 1,2,3,…,*G*
*p*	index for dyestuff product	*p* = 1,2,3…,*P*
*s*	index for suppliers	*s* = 1,2,3…,*S*
*i*	index for experts	*i* = 1,2,3,…,*I*
*o*	index for objectives	*o* = 1,2,3,…,*O*
*f*	index for green factors	*f* = 1,2,3,…,*F*

#### 3.4.2 Decision variables

**Table:** 

*Z*_*psg*_	$=\left\{\begin{array}{cc} 1 & \mathrm{if}\;\mathrm{product}\;p\;\mathrm{is}\;\mathrm{supplied}\;\mathrm{by}\;\mathrm{supplier}\;s\;\mathrm{to}\;\mathrm{GTSME}\;g\\ 0 & \mathrm{otherwise} \end{array}\right\}$
*Q*_*psg*_	Quantity of product *p* supplied by the supplier *s* to GTSME *g*

#### 3.4.3 Parameters

**Table:** 

***D***_***pg***_	demand for the product *p* by GTSME *g* (Kgs)
*C*_*sp*_	the production capacity of supplier *s* for the product *p* (Kgs)
*BS*_*ps*_	batch size of the supplier *s* for the product *p* (Kgs)
*P*_*ps*_	price of the product *p* supplied by the supplier *s* ($)
*PT*_*ps*_	production time of batch of the product *p* supplied by the supplier *s* (hrs)
*h*_*sg*_	distance between the supplier *s* and GTSME *g* (Km)
*LC*	labor cost ($)
***EC***	energy cost ($)
***IC***_***ps***_	quality inspection cost of the product *p* supplied by the supplier *s* ($)
***IT***_***ps***_	quality inspection time of product *p* supplied by the supplier *s* ($)
***qc***_***ps***_	quality complaints of product *p* sold by the supplier *s* during the last year
***TC***	transportation cost ($/Km)
*CE*_*h*_	carbon emissions tax for handling the product *p* ($/Kg)
*CE*_*t*_	carbon emissions tax during transportation of product *p* ($/Km)
***U***_***ps***_	number of units of product *p* sold by the supplier *s* in the last year
***aql***_***ps***_	acceptance quality limit of the product *p* by the supplier *s*
***AQL***	Standard Acceptance Quality Limit or Level
***v***	average speed (Km/hr)
***E***_***i***_	the experience level of the expert *i*
***IE***_***i***_	importance of the opinion of the expert *i*
***w***_***oi***_	weightage recommended for objective *o* by expert *i*
***θ***_***o***_	satisfaction level of objective *o*
***δ***_***ps***_	fuzzy deviation variable
*AFN*_*o*_	aggregate fuzzy number of objective *o*
***NFW***_***o***_	normalized fuzzy weight of objective *o*
***S***_***sp***_	the green score of supplier *s* for producing the product *p*
***R***_***sp***_	the rank of supplier *s* for producing the product *p*
***w***_***f***_	weight of each green factor *f*
***G***_***f***_	green factor *f*

#### 3.4.4 Objective functions

The following section describes the multiple objective functions and constraints used to model the defined problem mathematically.

*3.4.4.1 Green appraisal score of suppliers*. The green appraisal score is the total sum of the scores of the environmental parameters used to evaluate the suppliers. The green appraisal score is formulated as below:

$$\mathrm{Maximize}\;F_{green}=\sum_{s}\sum_{p}[S_{sp}\times Z_{psg}]$$ (1)

where

$$S_{sp}=\sum w_{f}G_{f}$$ (2)

After the formulation of the green score *S*_*sp*_ in Eq 2, the suppliers’ ranking is done to select the best supplier(s).

*3.4.4.2 Cost of the textile supply chain*. This objective function incorporates various costs that are incurred by the GTSME while their outsourcing process. The following equation describes the cost function:

$$\mathrm{Minimize}\;F_{cost}=\sum_{s}\sum_{p}(P_{ps}+LC+EC+CE_{h})\times Q_{psg}+\sum_{s}\sum_{p}[(TC+CE_{tr})\times d_{sg}+IC_{ps}]\times Z_{psg}$$ (3)

The first term of the equation describes the price of the product, labor, and energy cost, and carbon emission cost for handling the product. The second term gives the sum of transportation cost and carbon emission tax during transportation of the product, and the quality inspection cost.

*3.4.4.3 Quality of the product*. The proposed model takes customer quality complaints as a measure of the quality level of suppliers. This indirectly relates to the performance history of suppliers and their previous relationships with the customers. The quality function is described as follows:

$$\mathrm{Minimize}\;F_{quality}=\sum_{s}\sum_{p}[(qc_{ps}/U_{ps}x\;(1\;million)]\times Z_{psg}{{}_{}}_{}$$ (4)

The number of quality complaints is taken as per million units sold to the organization and are minimized for the improvement of quality. The quality complaints mentioned in the above expression are highly uncertain therefore fuzzy theory is used to handle them. The method of the fuzzy theory is shown below:

### Step 1: Fuzzy Membership Function

The quality complaints of the suppliers for the last year are considered uncertain therefore they are taken as a triangular fuzzy number. The general expression of a membership function for a triangular fuzzy number is shown in Eq 5 where *x* is the uncertain variable and *y*_1_, *y*_2_ and *y*_3_ are the boundary parameters.


$$\mu_{\Pi }(x)=\{\begin{array}{cc} \frac{x-y_{1}}{y_{2}-y_{1}} & if\;y_{1}\leq x\leq y_{2}\\ \frac{y_{3}-x}{y_{3}-y_{2}} & if\;y_{2}\leq x\leq y_{3}\\ 0 & otherwise \end{array}\}$$ (5)

Using the Eq 5, the membership function for the quality complaints is shown below, where *y*_1_ = *qc*_*ps*_−*δ*_1*ps*_; *y*_2_ = *qc*_*ps*_; *y*_3_ = *qc*_*ps*_+*δ*_2_:

$$\mu_{qc_{ps}}(x)=\{\begin{array}{cc} \frac{x-(qc_{ps}-\delta_{1ps})}{qc_{ps}-(qc_{ps}-\delta_{1ps})} & if\;qc_{ps}-\delta_{1ps}\leq x\leq qc_{ps}\\ \frac{qc_{ps}+\delta_{1ps}-x}{qc_{ps}+\delta_{2ps}-qc_{ps}} & if\;qc_{ps}\leq x\leq qc_{ps}+\delta_{2ps}\\ 0 & otherwise \end{array}\}$$ (6)

### Step 2: Fuzzification

Fuzzification is the technique of transforming a crisp function into a fuzzy function after determining the membership function. The quality objective in Eq 4 is a crisp function with an uncertain variable of quality complaints. This objective can be changed to its equivalent fuzzy function as shown below:

$${\left[F_{quality}\right]}^{Fuzzy}=d(F_{quality},\;0_{1})=\sum_{s}\sum_{p}[\frac{d(qc_{ps},\;0_{1})}{Rq_{ps}}\times (1\;million)]\times Z_{psg}$$ (7)

### Step 3: Defuzzification

Defuzzification is the process of converting the fuzzy function back to its equivalent crisp function for performing further operations. There are various methods present in literature for defuzzification such as the center of the largest area, the centroid method, center of sums, signed distance method, first or last maxims, and many more. The Signed-distance method is clear, easy, and outperforms in many complex conditions [114]. Therefore, the equivalent defuzzified function using the signed distance method is shown as follows:

$$d(qc_{ps},\;0_{1})=\frac{\left(qc_{ps}-\delta_{1ps}+2qc_{ps}+qc_{ps}+\delta_{2ps}\right)}{4}=\frac{4qc_{ps}+(\delta_{2ps}-\delta_{1ps})}{4}$$ (8)

Using Eq 8 in the objective function of quality mentioned in Eq 7 to get the final crisp objective function of quality that can be utilized in the proposed framework:

$${\left[F_{quality}\right]}^{Crisp}=\sum_{s}\sum_{p}[\frac{4\;qc_{ps}+(\delta_{2ps}-\delta_{1ps})}{4Rq_{ps}}\times (1\;million)]\times Z_{psg}$$ (9)

*3.4.4.4 Time*. Total time is the sum of time required for production, transportation, and quality inspection of a product. The objective function for time is formulated as below:

$$\mathrm{Minimize}\;F_{time}=\sum_{s}\sum_{p}[\frac{PT_{ps}}{BS_{ps}}\times Q_{psg}]+\sum_{s}\sum_{p}[\frac{h_{sg}}{v}+IT_{ps}]\times Z_{psg}$$ (10)

Where the first term describes the production time of the product taken by suppliers. The second term describes the transportation time between the suppliers and the purchaser i.e. GTSME, and quality inspection time.

Eqs 1–4 and 10 show the objective functions of the model. These objective functions are subjected to the constraints discussed in the next section.

#### 3.4.5 Constraints


$$\sum \;Q_{psg}=D_{pg}\;\forall_{p};\forall_{g}$$ (11)

$$Q_{psg}\leq C_{ps}\times Z_{psg}\;\forall_{p};\forall_{s};\forall_{g}$$ (12)

$$aql_{ps}\times Z_{psg}\leq AQL\;\forall_{p};\forall_{s};\forall_{g}$$ (13)

$$R_{sp}\times Z_{psg}\leq n\;\forall_{p};\forall_{s};\forall_{g}$$ (14)

$$Q_{fsj}\geq 0$$ (15)

$$Z_{psg}Є\;\{0,1\}$$ (16)

Eqs 11–16 show the constraints subjected to these objective functions. Eq 11 is the demand constraint which shows that the sum of quantity allocated to the suppliers is equal to the demand of the GTSME. Eq 12 is the capacity constraint which shows that the quantity allocated to the suppliers is less than or equal to the production capacity of the suppliers. Eq 13 ensures the required Acceptance Quality Limit (AQL) of the suppliers whereas Eq 14 describes the green score ranking constraint which shows that the top ‘n’ suppliers having the highest green score must be selected. In this constraint, the number of suppliers to be selected is completely subjective according to industry. Eqs 15 and 16 are the non-negativity constraints showing that the quantity is always greater than zero, and the binary variable Z is 1 when the quantity is supplied by the supplier(s) and 0 otherwise.

### 3.4.6 Proposed hybrid framework for green supplier selection

This section describes the proposed MCDM model including QFD and multi-objective interactive fuzzy weighted programming technique. The QFD technique is used to find the green factors’ weights to know which of the parameters have more importance for the stakeholders of the subject industry. Also, the QFD technique helps in finalizing the weights of stakeholders’ requirements for the green supplier selection problem. Two HOQs have been developed in the model for aligning the stakeholders’ requirements with the green factors resulting in a convenient supplier selection technique for the industry. Although the proposed model is a multi-objective problem and all objectives are equally weighted, but these weights can be varied from industry to industry. For example, in the textile industry, according to experts, the importance of quality and time is greater than the cost of the product. In Green Textile Supply Chains (GTSCs), the experts rank green objective function as the main concern to promote sustainability and reduce environmental deterioration. After determining the Fuzzy preference weights of the four objective functions, the interactive multi-objective fuzzy programming technique is utilized to find an optimal solution set. The stepwise solution method is mentioned in the next sections.

*3.4.6.1 Green factors rating for the suppliers*. First of all, the scores of the green parameters are found through interviews with each supplier. Each parameter is important for environmental concerns during the manufacturing of dyestuff products. These green scores help to compare the suppliers considering their production processes and other operational activities. The scores are ranged from 1 to 4 corresponding to the environmental scale of green parameters. These scores are shown in Table 2. These individual scores of all parameters are summed up again to calculate the scores of green factors i.e. green design, green logistics, and EMS. Table 3 shows the respective scores of the green factors for five different suppliers.

**Table 2:** Scores of green parameters.

Suppliers	Green Packaging	Energy & Natural Resource Consumption	Degree of use of environment-friendly materials	GHG Emissions during transportation & Product handling	Air Pollution Control during transportation	Degree of having ISO or other Environmental Certifications	Degree of having Environmental Plans & Policies	Solid Waste Treatment	Waste Water Treatment
1	3	2	4	3	4	3	3	3	2
2	2	3	3	3	2	3	4	1	3
3	4	1	3	2	3	2	3	2	2
4	3	3	2	4	3	3	2	3	4
5	2	3	4	4	3	2	3	1	4

**Table 3:** Scores of green factors.

Suppliers	Green Design	Green Logistics	Environmental Management System
1	9	7	11
2	8	5	11
3	8	5	9
4	8	7	12
5	9	7	10

*3.4.6.2 Quality function deployment (QFD) for the model*. QFD technique is a useful tool for transforming the customer requirements into technical specifications. It is a good tool for organizations that focus on tuning the voice of clients and fulfilling their requirements. QFD method is used to develop HOQ for solving the problem. This technique considers the customer requirements as “Whats” and the design features or technical specifications as “Hows”. The main body of the house is the correlation matrix of “Hows” with each of the “Whats”. The ranking between these two can be done using the values of 0, 3, 6, and 9 showing the weak, moderate, and strong relationships respectively. The total of these values in every column is a relative importance rating of each technical specification. This information is valuable for positioning each of the "Hows" and to choose where to designate the greater part of the assets. The HOQ matrix contains the Whats, Hows, the interrelationship matrix between Whats and Hows, weights of Whats, and weights of Hows [115].

QFD technique is applied to find the individual weights of the above-mentioned green factors. In the proposed model, two HOQs have been developed. HOQ1 links the stakeholders of the company (the most important departments associated with the supplier selection process) with their requirements. This HOQ gives us the individual weights of each stakeholder requirement. The stakeholder requirements are shown in Table 4. The stakeholders are considered as Whats and their requirements are considered as Hows in the first HOQ. HOQ2 links the stakeholder requirements with the three green factors and gives us the final weight of each factor ready to be used in the objective function. The stakeholder requirements are considered as the Whats and the green factors are considered as the Hows in the second HOQ. Table 4 (HOQ1) and Table 5 (HOQ2) show the formation or block diagram of the HOQ matrix of the proposed model.

**Table 4:** House of quality 1.

Stakeholders					Stakeholder Requirements							
	Importance Rating of Stakeholders	Compliance with Industrial Procedures	Compliance with Environmental Policies	Financial Stability		Quality Control System	Waste Disposal Program	Pollution Control	Total Cost Ownership	Reliability of Order Fulfilment	Reverse Logistics	Reference from Satisfied Customers
Finance	0.167			9		3			9	6		3
Procurement	0.300	3	3	6		6	3	3	6	9	3	9
Production	0.167	9	6	3		6	6	6	6	6	6	3
Quality Control	0.167	9	6			9	6	6			6	6
Health, Safety & Environment	0.200	6	9			6	9	9			9	
Importance Rating of Parameters	46.545	5.106	4.704	3.804		6.006	4.704	4.704	4.305	4.704	3.804	4.704
		0.110	0.101	0.082		0.129	0.101	0.101	0.0925	0.101	0.082	0.101

**Table 5:** House of quality 2.

Stakeholder Requirements	Green Parameters			
	Weight	Green Design	Green Logistics	EMS
Compliance with Industrial Procedures	0.110	9	6	3
Compliance with Environmental Policies	0.101	9	9	9
Financial Stability	0.082	6	3	3
Quality Control System	0.129	6	3	9
Waste Disposal Program	0.101	6	6	9
Pollution Control	0.101	6	9	6
Total Cost Ownership	0.0925	6	6	3
Reliability of Order Fulfilment	0.101			3
Reverse Logistics	0.082	9	6	9
Reference from Satisfied Customers	0.101	3	3	
Importance Rating of Parameters	16.5195	5.973	5.067	5.4795
		0.36	0.31	0.33

*3.4.6.3 α-Extreme solutions*. Each objective function is solved individually to find the α-extreme solutions which are used as constraint functions in the final solution of the problem. The lower and upper bounds of that objective function are decided by the decision maker’s intervention and the same is done for all the other three objective functions. All the α-extreme solutions including the lower and upper bounds are recorded to develop a pay-off table mentioned in section 4.2.

*3.4.6.4 Linearization of objective functions*. The objective functions are linearized by using a fuzzy membership function. Here, a triangular membership function is used for linearization. The following Eq 17 shows the generic form of triangular membership function:

$$\theta_{o}=\left\{\begin{array}{cc} 0 & f\geq {f_{o}}^{\alpha -lb}\\ \frac{\left({f_{o}}^{\alpha -ub}-f\right)}{\left({f_{o}}^{\alpha -ub}-{f_{o}}^{\alpha -lb}\right)} & {f_{o}}^{\alpha -lb}<f<{f_{o}}^{\alpha -ub}\\ 1 & f\leq {f_{o}}^{\alpha -ub} \end{array}\right\}$$ (17)

Where ${f_{o}}^{\alpha -lb}$ and ${f_{o}}^{\alpha -ub}$ are the lower and upper bounds of objective function ‘o’

*3.4.6.5 Finding weights using fuzzy linguistic scale*. Normalized priority weights are found for every objective to transform the multi-objective problem into a single-objective interactive fuzzy weighted problem. For this, a fuzzy linguistic scale is utilized in which the most important variables are close to 1 and the least important is close to 0. Table 6 shows the fuzzy linguistic scale. A group of experts rates the weights according to their opinion. According to previous research in this field of study, the opinions of all experts are weighted equally. However, in the proposed research the experts have different managerial skills and experience; therefore, giving equal weightage to all experts is not an effective method of decision-making. Aggregate Fuzzy Number (AFN) is established from the following calculations using the experience of experts:

### *a)* Importance of opinion of Expert


$$IE_{i}=E_{i}/IE_{i}$$ (18)

Eq 18 is used to compute the significance of the opinion of expert ’i’ comparative with all experts. The AFN is calculated by Eq 19 using the importance of opinions of experts. Final Normalized Fuzzy Weight (NFW) of the objective ‘o’ is calculated using Eq 20.

### *b)* Aggregate Fuzzy Number

$$AFN_{o}=\frac{IE_{i}\times w_{oi}}{I}$$ (19)

### *c)* Normalized Fuzzy Weights of Objectives


$$NFW_{o}=\frac{AFN_{o}}{\sum_{o}AFN_{o}}$$ (20)

**Table 6:** Fuzzy linguistic scale.

Importance of Objective	Fuzzy numbers
Least Important	(0.0,0.1,0.2)
Less Important	(0.2,0.3,0.4)
Important	(0.4,0.5,0.6)
More Important	(0.6,0.7,0.8)
Most Important	(0.8,0.9,1.0)

After finding the α-extreme solution and NFW for each objective function, the interactive fuzzy goal programming technique is used to solve the model. The final multi-objective problem for green supplier selection becomes:

$$\mathrm{Maximize}\;F=NFW_{o}\times \theta_{o}$$ (21)

Subject to constraints in Eqs 11–16.

## 4. Application of model

The following section shows the validity and practical application of our multi-objective green supplier selection problem. This model considers a set of 5 suppliers and a GTSME (buyer or consumer of the dyestuff provided by the suppliers). The GTSME has the aim to select the best supplier from 5 available suppliers for each product using the selection criteria of green appraisal score, cost, quality, and time. The objectives of the selection of suppliers are to maximize the green appraisal score and select the top 3 suppliers with the highest green score along with minimizing cost, quality complaints, and lead time of the supply chain.

### 4.1 Numerical experiment

This section shows the data sets of various parameters to be used for the numerical analysis of the proposed model. The demand for the GTSME is a constant value i.e 15239 kgs. The production capacity, production time, batch size, and product price of the suppliers are given in Table 7. The quality complaints, the number of units sold for the last year, and the acceptance quality limit (aql) are given in Table 8. The distances between suppliers and GTSME are given in Table 9. Other parameters like transportation cost, energy cost, labor cost, carbon emissions tax for handling and transportation, average speed, transportation cost, quality inspection cost, and time and standard Acceptance Quality Level (AQL) value have been shown in Table 10.

**Table 7:** Capacity, production time, batch size, and price of the product of each supplier.

Suppliers	Production capacity in Kgs	Production time of supplier in hrs.	Batch size of the supplier in Kgs	Price of the product given by the supplier in $
Supplier 1	20000	7	900	3.2
Supplier 2	16000	9	800	3.7
Supplier 3	21000	8	1000	2.9
Supplier 4	23000	7	1100	4.1
Supplier 5	22000	8	1500	3.2

**Table 8:** Quality complaints, number of units sold, and aql of each supplier.

Suppliers	Quality complaints in the last year	Number of units in the last year	AQL of supplier
Supplier 1	143390	230	3.2
Supplier 2	126845	142	2.9
Supplier 3	228321	97	3.3
Supplier 4	104785	201	1.5
Supplier 5	131257	187	3.3

**Table 9:** Distance between suppliers and buyer in Km.

Suppliers	Distance
Supplier 1	32
Supplier 2	43
Supplier 3	41
Supplier 4	36
Supplier 5	12

**Table 10:** Other parameters used in the model.

Other Parameters	Values	Units
Demand of buyer	15239	Kgs
Standard Acceptance Quality Limit (AQL)	3.5	-
Inspection Time	2	hrs
Inspection Cost	15	$
Energy Cost	1	$
Labor Cost	5	$
Carbon Emissions Tax during Handling of Product	0.2	$
Carbon Emissions Tax during Transportation	9x10^-4^	$
Transportation Cost	0.05	$
Average Speed	60	Km/hr
δ_1ps_	20	-
δ_2ps_	40	-

### 4.2 Solution

The numerical example was solved using MATLAB (R2021a) on a personal computer with 12GB RAM and a processor of 2.4 GHz. The detailed solution approach provided in section 3.4.6 is applied to solve the problem using a branch and bound algorithm.

A stepwise solution of numerical assessment is given below:

1. In the first step, the scores of green parameters are used in the objective of the green appraisal score, and all suppliers are evaluated for the green score. When the score is calculated, the suppliers are ranked based on the highest scores. The Green Score objective is set as a constraint for the next 3 objectives and the optimal solution is found.
2. The deterministic model was solved including all the objectives using Interactive Multi-Objective Fuzzy Linear Programming. The first three objective functions are minimized separately while the last objective function *F*_*green*_ is maximized for the green score ranking. The optimal solutions of each objective function are recorded and displayed in Table 11.

**Table 11:** Optimal solutions of objective problems.

Objective Functions	Optimal Solutions
*F*_*cost*_($)	143,260
*F*_*quality*_(partsppm)	21,062,790
*F*_*time*_(hrs)	83.4747
*F*_*green*_	6

3. In the next step, the bounds of each objective are found to form a payoff table. For obtaining this payoff table as shown in Table 12, all the objective functions are solved and optimized separately, and then the value is set as a constraint while optimizing the other objective functions. For example, the first row and first column of the payoff table show the optimal value of cost which is computed using the following steps:

**Step 1:** Minimize *F*_*cost*_

As the optimal value ***F***_***cost***_ is calculated, it is set as a constraint for the next objectives.

The value of quality in the second column and the first row of the payoff table is found by the following problem:

**Step 2:** Minimize *F*_*quality*_ subject to *F*_*cost*_ = 143.260 *and F*_*green*_ = 6

Similarly, in the third column and the first row of the payoff table, the optimal value of time is found by the following problem:

**Step 3:** Minimize*F*_*time*_ subject to *F*_cos*t*_ = 143,260 *and F*_*green*_ = 6 *F*_*cost*_ = 143,260 *and F*_*green*_ = 6

Similarly, in the fourth column and first row of the payoff table, the value of the green appraisal score rank is found by the following problem:

**Step 4:** Minimize*F*_*green*_ subject to *F*_cos*t*_ = 143,260 *and F*_*green*_ = 6 *F*_*cost*_ = 143,260 *and F*_*green*_ = 6

The above four steps are also subjected to constraints (11)-(16). These steps are repeated for quality, time, and green score rank to obtain the payoff table shown below:

**Table 12:** Pay-off table of objective functions.

POT	*F*_*cost*_	*F*_*quality*_	*F*_*time*_	*F*_*green*_
*F*_*cost*_($)	**143,260**	24,545,994	83.4747	3
*F*_*quality*_(partsppm)	156,980	**21,062,790**	99.5755	2
*F*_*time*_(hrs)	143,260	24,545,994	**83.4747**	3
*F*_*green*_	143,300	78,589,634	125.8589	**6**

4. The subsequent stage is to compute a fuzzy membership function for all objectives to ascertain the satisfaction level of every objective. The fuzzy membership function of every objective is computed using Eq 5. Eqs 22–25 are the required satisfaction levels of cost, quality, time, and green score rank objectives.

$$\theta_{cost}=\left\{\begin{array}{cc} 0 & F_{cost}\geq 143,260\\ \frac{156,980-F_{cost}}{156,980-143,260} & 143,260<F_{cost}<156,980\\ 1 & F_{cost}\leq 156,980 \end{array}\right\}$$ (22)

$$\theta_{quality}=\left\{\begin{array}{cc} 0 & F_{quality}\geq 21,062,790\\ \frac{78,589,634-F_{quality}}{78,589,634-21,062,790} & 21,062,790<F_{quality}<78,589,634\\ 1 & F_{quality}\leq 78,589,634 \end{array}\right\}$$ (23)

$$\theta_{time}=\left\{\begin{array}{cc} 0 & F_{time}\geq 83.4747\\ \frac{125.8589-F_{time}}{125.8589-83.4747} & 83.4747<F_{time}<125.8589\\ 1 & F_{time}\leq 125.8589 \end{array}\right\}$$ (24)

$$\theta_{green}=\left\{\begin{array}{cc} 0 & F_{green}\geq 2\\ \frac{6-F_{green}}{6-2} & 3<F_{green}<6\\ 1 & F_{green}\leq 6 \end{array}\right\}$$ (25)

5. NFWs for all the objective functions are determined by the use of the fuzzy linguistic scale in Table 6. Five experts with different levels of managerial skills having respective experiences in the procurement process are selected from the textile supply chain to assign the weights to the objective functions. Eq 18 is used to calculate the importance given to every expert. For example, for expert i = 1, IE_1_ = 25/ (25+10+35+44+50) = 0.152439. The importance given to other experts is shown in Table 13. Each expert prioritized the objective functions according to their experience, and this linguistic value is decoded using Table 6. For example, the first expert considered cost as most important so the value corresponding to most important is 0.9 which is the average of (0.81, 0.9, 1). Using Eq 19, the aggregate fuzzy number of cost is calculated as shown below:

$$AFN_{cost}=\frac{\left(0.152439\times 0.9+0.060976\times 0.7+0.213415\times 0.5+0.268293\times 0.3+0.304878\times 0.3\right)}{5}AFN_{cost}=0.091707$$

**Table 13:** Determination of normalized fuzzy weights using experts’ opinion.

Experts	Relative importance grade of expert (0–50)	The relative weight of expert	Objective importance preference of each expert committee member			
			Cost	Quality	Time	Green
1	25	0.152439	most important	more important	less important	most important
2	10	0.060976	more important	least important	most important	important
3	35	0.213415	important	most important	more important	most important
4	44	0.268293	less important	most important	more important	more important
5	50	0.304878	less important	most important	less important	most important

Similarly, the aggregate fuzzy number of the other three objective functions are calculated. The normalized fuzzy weights of all objective functions are determined by Eq 20. The normalized fuzzy weight for the cost is calculated as mentioned below:

$$NFW_{cost}=\frac{0.091707}{\left(0.091707+0.164146+0.105854+0.164390\right)}NFW\;\cos t=0.174$$

Similarly, *NFW*_*quality*_ = 0.312; *NFW*_*time*_ = 0.201; *NFW*_*green*_ = 0.312

Using the above-mentioned weights and the Eqs (16–19), the multi-objective problem is converted to a single objective problem:

$$\mathrm{Minimize}\;f=NFW_{cost}\times \theta_{cost}+NFW_{quality}\times \theta_{quality}+NFW_{time}\times \theta_{time}+NFW_{green}\times \theta_{green}$$ (26)

### 4.3 Computational results

**Fig 2:**
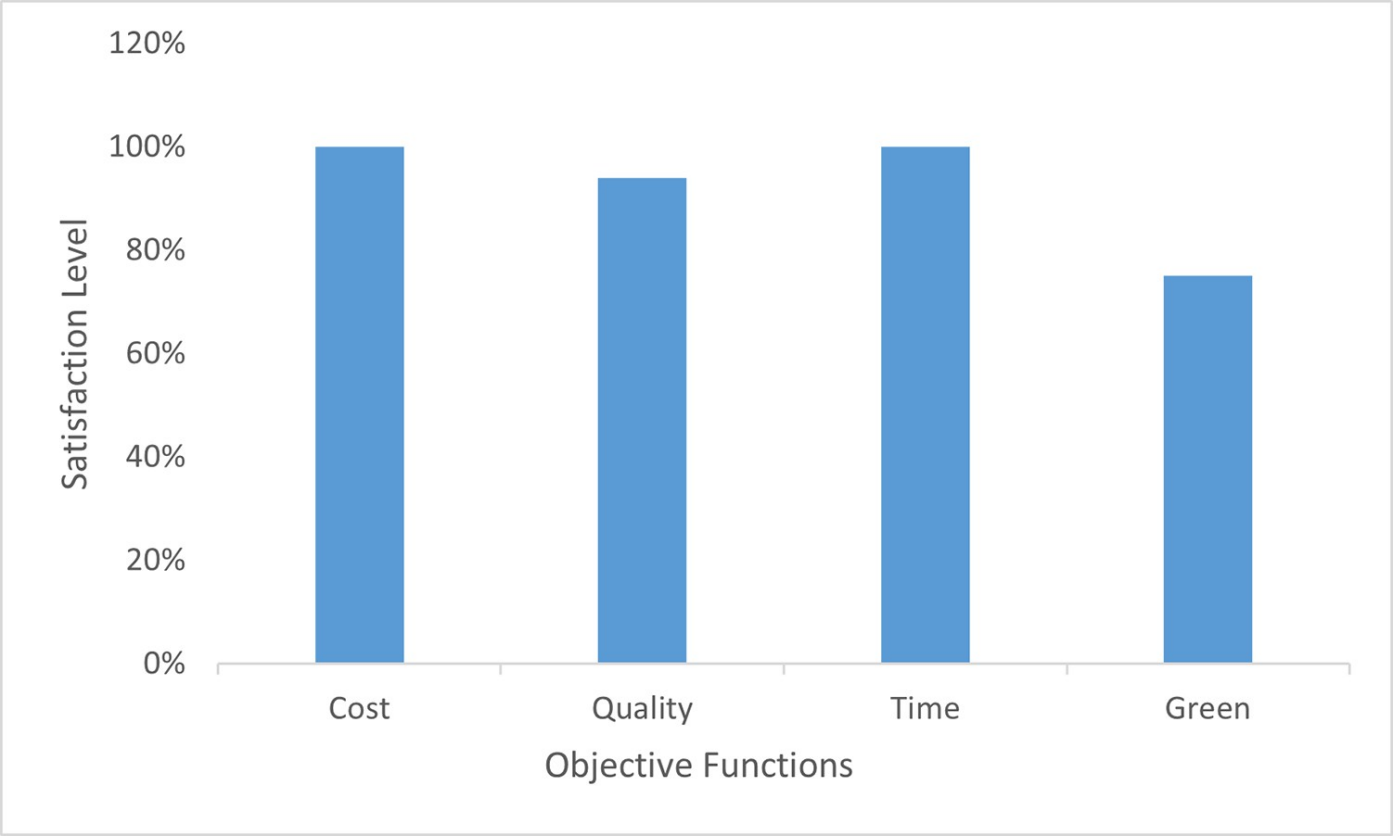
Achieved satisfaction levels.

Table 14 shows the optimal solutions and trade-off for the satisfaction levels between all the conflicting objectives of the model based on the opinions of experts. Fig 2 shows the achieved satisfaction levels of all objective functions. Table 15 shows the qualified supplier(s) and the quantity of order/demand allocated to each supplier based on the demand and capacity constraints; in the textile supply chain network. According to Table 15, Supplier 5 is qualified in the multi-objective problem and the quantity allocated to this supplier is equal to the demand of the GTSME.

**Table 14:** Optimal solutions to objective functions.

Objective Functions	Optimal Solutions	Satisfaction Level
*F*_*cost*_($)	143,260	100%
*F*_*quality*_(partsppm)	24,546,000	93.9451%
*F*_*time*_(hrs)	83.4747	100%
*F*_*green*_	3	75%

**Table 15:** Qualified supplier(s).

Suppliers	Green Score Ranking		Cost		Quality		Time		Multi-Objective Fuzzy Weighted Programming	
	Z1	Q1	Z2	Q2	Z3	Q3	Z4	Q4	Z	Q
Supplier 1	1	15239	0	0	0	0	0	0	0	0
Supplier 2	0	0	0	0	0	0	0	0	0	0
Supplier 3	0	0	0	0	0	0	0	0	0	0
Supplier 4	1	0	0	0	1	15239	0	0	0	0
Supplier 5	1	0	1	15239	0	0	1	15239	1	15239

### 4.4 Analysis and discussion

Results of the evaluation of the proposed model verify a trade-off between all the objective functions. We can observe from Table 14 that the optimal solutions of all objectives are between the upper and lower bounds, which shows the robustness of the model. The objective functions of cost and time have a 100% satisfaction level whereas the objective function of quality has the satisfaction of 93.95% and the green objective has a satisfaction level of 75% which shows that there is a tradeoff between the importances of all these objectives according to the experts. The tradeoff occurs due to the difference between the economic and environmental concerns of the stakeholders of the textile supply chain.

#### 4.4.1 Satisfaction level

It is the percentage value of the deviation of an optimal solution of a function from its lower and upper bounds. According to Table 12, the optimal value of cost is 143,260, and the satisfaction level of cost in Eq 17 is computed as:

$$\theta_{cost}=\frac{156,980-143,260}{156,980-143260}=100\%$$ (27)

This shows that the closer the optimal value to the lower bound, the highest is the satisfaction level of that objective function, and vice versa. In the ideal case, a 100% satisfaction level is achieved for an objective but due to the conflicting objectives, a tradeoff between the satisfaction levels is observed. The lower and upper bounds of an objective function depending on the values and constraints of the other objectives. In the proposed model, the satisfaction levels of quality and green score rank are less than 100% due to the conflicting nature of other objectives.


$$\theta_{cost}=\frac{156,980-143,260}{156,980-143260}=100\%$$

#### 4.4.2 Constraints satisfaction

The optimization approach provides optimal solutions to the defined objectives. The precision of the optimization problem lies in the fulfillment of constraints along with the optimal values. The most important constraints are the demand and capacity constraints which need to be fulfilled to successfully perform order quantity allocation. The demand for GTSME in our model was 15239 Kgs. As shown in Table 15, the quantity allocated to the qualified supplier is 15239 Kgs. Hence, it shows that the demand constraint is satisfied. Similarly, the quantity gets divided into selected suppliers if the capacity of any qualified supplier is less than the demand. Table 16 shows the allocation of quantity to multiple suppliers when the capacity of suppliers is less than the demand for GTSME. Similarly, the model satisfies the green constraint by selecting the top three suppliers in the green score objective. As shown in Table 16, the top three suppliers based on the green score are Supplier 1, 4, and 5.

**Table 16:** Demand and capacity constraints satisfaction.

Suppliers	Green Score Ranking		Cost		Quality		Time		Multi-Objective Fuzzy Weighted Programming	
	Z1	Q1	Z2	Q2	Z3	Q3	Z4	Q4	Z	Q
Supplier 1	1	10000	1	4239	0	0	0	0	0	0
Supplier 2	0	0	0	0	0	0	0	0	0	0
Supplier 3	0	0	0	0	0	0	0	0	0	0
Supplier 4	1	5239	0	0	1	12000	1	11000	1	11000
Supplier 5	1	0	1	11000	1	3239	1	4239	1	4239

#### 4.4.3 Managerial insights

In the proposed problem, the GTSME intends to choose the most suitable supplier(s) based on a business quartet of cost, quality, time, and green score rank. This model can be easily used by the textile supply chain experts for optimal decision-making in the procurement of dyestuff products. The introduction of human opinions regarding the importance of objectives makes the model more realistic. The managers can easily prioritize according to their preferences and make optimal decisions. The first three objectives are economic whereas the green score deals with environmental concerns, which nowadays is extremely important for industrial supply chains.

The components of the business quartet (cost, quality, time, and green score ranking) are all conflicting objectives and require a trade-off among them to provide a satisfactory solution to the GTSME’s problem. Each objective function has to compromise to incorporate the importance of other objectives. The numerical tables provided above show that the satisfaction levels of cost and time are 100% whereas the satisfaction level of green score ranking is 75% and for quality, it is 93.95% which shows that the green objective has to compromise more to accommodate other objectives. These satisfaction levels can be varied by changing the importance weights of the objectives according to experts. Eq 28 shows the achievement of every objective according to the satisfaction levels of these objectives. The percentage goal achieved by each objective can be found by:

$$PercentageGoalofeachobjectiveGoal_{o}=\frac{\theta_{o}}{\sum \theta_{o}}x100$$ (28)

The above provides the percentage goal achievement of each objective which are:

$$PercentageGoal_{\cos t}=27.10\%;\;PercentageGoal_{quality}=25.46\%;\;PercentageGoal_{time}=27.10\%;\;PercentageGoal_{green}=20.3\%$$

**Fig 3:**
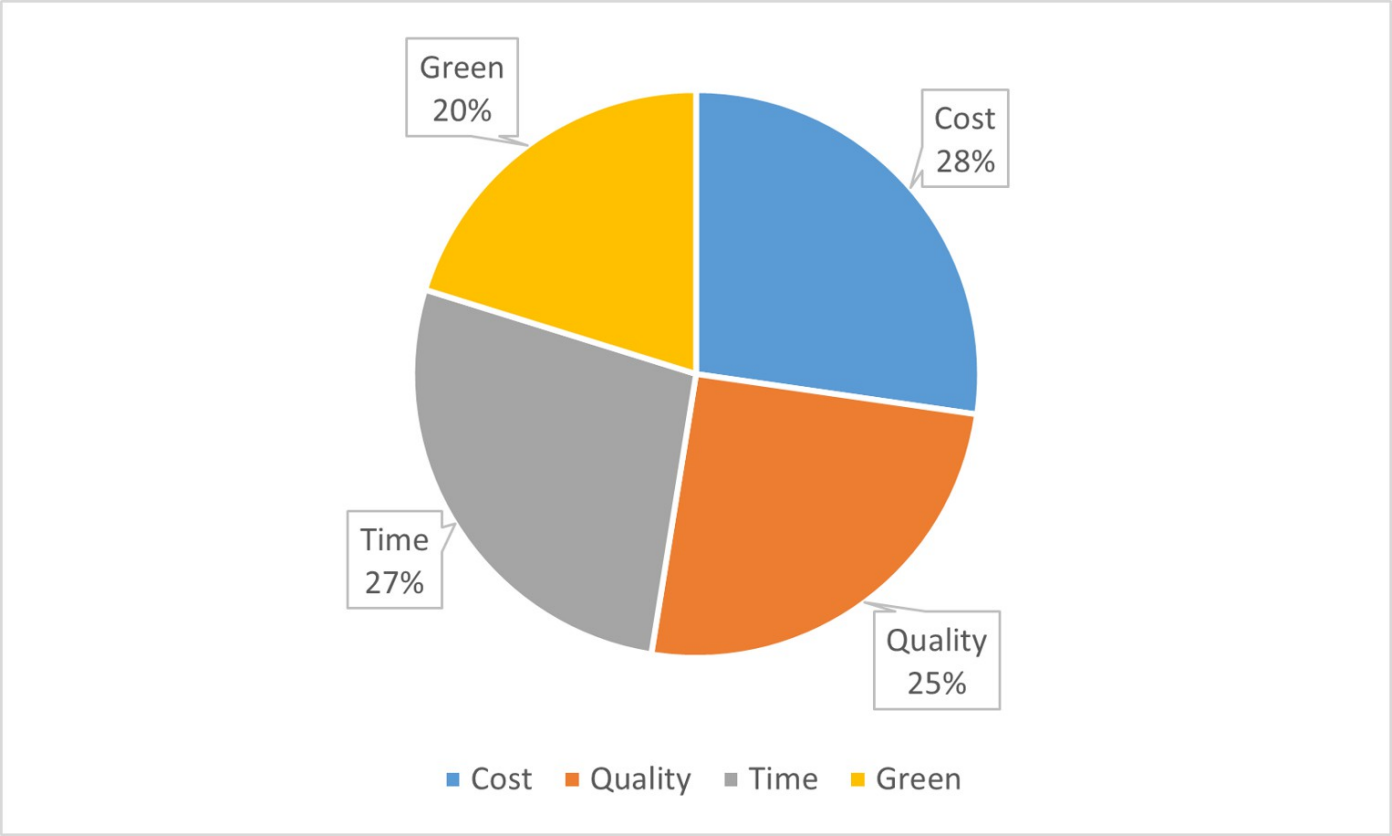
Contributors of the business quartet.

Fig 3 describes the contribution of every objective of the model.

#### 4.4.4 Effect of variation in uncertainty

In this research, the quality complaints for the last year (which are used in the quality objective) are taken as highly uncertain, and fuzzy theory is used to incorporate them into the crisp model. The quality complaints are taken as a triangular fuzzy number. The triangular membership function is used and the defuzzification to the crisp model is done through the signed distance method. Here, the model is analyzed with different values of uncertainty to see the effect of uncertainty on the model. First, the uncertainty is taken as zero which means the uncertainty is removed from the model and the quality complaints are taken as a crisp number. Afterward, as shown in Table 17, the values of uncertainty are added in the model to see the effect. Table 17 shows the effect of uncertainty.

**Table 17:** Effect of uncertainty.

Value of uncertainty	Objective Function	Value	Satisfaction Level
δ_1_ = 0; δ_2_ = 0	Cost ($)	143,260	100%
	Quality (complaints ppm)	521.32	86.3741%
	Time (hrs)	83.4747	100%
	Green score rank	6	75%
δ_1_ = 40; δ_2_ = 80	Cost ($)	143,260	100%
	Quality (complaints ppm)	24,547,000	93.9454%
	Time (hrs)	83.4747	100%
	Green score rank	6	75%

The model shows a variation in the quality objective by changing the model from a crisp one to an uncertain one, as mentioned in Table 17. When the model is crisp, i.e. δ_1_ = 0; δ_2_ = 0 then the satisfaction level of quality is 86.3741% whereas the satisfaction level increases when the uncertainty is increased to 93.95%. This shows that the model is well incorporating the change in uncertainty. The satisfaction level of a crisp model is less as complaints are generally considered to be uncertain. However, both models have their attributes according to the defined problem.

#### 4.4.5 Sensitivity analysis

*4*.*4*.*5*.*1 Sensitivity analysis for green scores*. The sensitivity analysis is performed by changing the scores of the green parameters of each supplier. By changing this information, the model can select different suppliers having similar data. This results in the incorporating of various managerial opinions in the model. Table 18 shows the new scores of each supplier. When these parameters are changed, the new total green score of each supplier is calculated, and hence the new suppliers are ranked accordingly. The suppliers that qualify the given green criteria are shown in Table 19.

**Table 18:** Scores of green parameters of suppliers after sensitivity analysis.

Suppliers	Green Packaging	Energy & Natural Resource Consumption	Degree of use of environment-friendly materials	GHG Emissions during transportation & Product handling	Air Pollution Control during transportation	Degree of having ISO or other Environmental Certifications	Degree of having Environmental Plans & Policies	Solid Waste Treatment	Waste Water Treatment
1	1	2	4	1	1	3	1	1	1
2	3	1	2	3	2	2	4	1	3
3	2	1	3	4	4	2	4	3	3
4	3	4	2	4	3	4	3	3	4
5	2	3	3	3	3	2	3	2	1

**Table 19:** Qualified supplier(s) after sensitivity analysis.

Suppliers	Green Score Ranking		Cost		Quality		Time		Multi-Objective Fuzzy Weighted Programming	
	Z1	Q1	Z2	Q2	Z3	Q3	Z4	Q4	Z	Q
Supplier 1	0	0	0	0	0	0	0	0	0	0
Supplier 2	0	0	0	0	0	0	0	0	0	0
Supplier 3	1	15239	1	15239	0	0	0	0	0	0
Supplier 4	1	0	0	0	1	15239	0	0	1	15239
Supplier 5	1	0	0	0	0	0	1	15239	0	0

*4*.*4*.*5*.*2 Sensitivity analysis for data parameters*. A sensitivity analysis is performed on all the key parameters used in the model. The parameters that are changed in the model are demand, price given by each supplier, production time, batch size, quality complaints, and units of each supplier. The changes in the values of all objective functions are noted in the Table 19. Following results are obtained by changing each parameter:

- The most influential parameter in the supply chain is the demand. When the demand is decreased, cost and time are decreased symmetrically but when the demand is increased, the cost and time are increased by higher values. This shows cost and time are more sensitive towards positive changes in demand. However, when product price is changed, a symmetric change is seen in cost.
- When production time of each supplier is changed, a symmetric change in time is seen. However, the other three objectives are seen unchanged.
- In case of batch size of each supplier, time is affected more in negative change as compared to positive change. When batch size is decreased, time is increased up to 97% whereas when the batch size is decreased, time is decreased up to 32%. This shows that time is more sensitive towards negative changes in batch size. The other objective functions remain unaffected.
- A symmetric change in the value of quality objective is observed when quality complaints and number of units are changed.

The model shows a variation in the values of the concerned objective functions by changing the key parameters of the model. Table 20 shows that the model is well incorporating the changes. Hence, the model can work on any data set from any industry applicable.

**Table 20:** Sensitivity analysis on MODM parameters.

Parameter	% Change in Parameters	% Change in *F*_*cost*_	% Change in *F*_*quality*_	% Change in *F*_*time*_
Demand ***D***_***pg***_	-50%	-50%	0%	-49%
	-25%	-25%	0%	-24%
	+25%	25%	0%	24%
	+50%	64%	0%	77%
Product price ***P***_***ps***_	-50%	-17%	0%	0%
	-25%	-9%	0%	0%
	+25%	9%	0%	0%
	+50%	17%	0%	0%
Production time ***PT***_***ps***_	-50%	0%	0%	-49%
	-25%	0%	0%	-24%
	+25%	0%	0%	24%
	+50%	0%	0%	49%
Batch size ***BS***_***ps***_	-50%	0%	0%	97%
	-25%	0%	0%	32%
	+25%	0%	0%	-19%
	+50%	0%	0%	-32%
Quality complaints ***qc***_***ps***_	-50%	0%	-50%	0%
	-25%	0%	-25%	0%
	+25%	0%	25%	0%
	+50%	0%	50%	0%
No. of Units ***U***_***ps***_	-50%	0%	-50%	0%
	-25%	0%	-25%	0%
	+25%	0%	25%	0%
	+50%	0%	50%	0%

#### 4.4.6 Performance evaluation of proposed methodology

The numerical example is solved using two different optimization techniques: weighted goal programming and epsilon constrained method. Multi-objective interactive fuzzy weighted programming and weighted goal programming techniques work by assigning weights to each objective whereas epsilon constrained method works by converting the objective problems into constraints. The values of each objective through different techniques are recorded in Table 21. To evaluate the performance of each methodology, we consider percentage gap in all the objectives. [109] used Eq 29 to find the percentage gap between each objective.


$$Gap=\frac{AV-BV}{AV}\times 100$$ (29)

**Table 21:** Comparison of different optimization methods.

Optimization Method	Objectives			
	*F*_*cost*_	*F*_*quality*_	*F*_*time*_	*F*_*green*_
Goal Programming	143,300	78,589,634	125.859	6
Epsilon Method	332,260	78,589,634	88.608	1
Multi-objective Interactive Fuzzy Weighted Programming	143,260	24,546,000	83.474	3

The percentage gaps for each objective are shown in Table 22. For the first objective i.e. cost, the best value is 143,260 so percentage gap for the multi-objective interactive fuzzy weighted programming is zero. Similarly, the percentage gap for each objective function of other methodologies is calculated.

**Table 22:** Percentage gap in objectives for different optimization methods.

Optimization Method	Objectives				Cumulative gap
	*F*_*cost*_	*F*_*quality*_	*F*_*time*_	*F*_*green*_	
Goal Programming	0.020%	220.000%	50.775%	500.000%	770.79%
Epsilon Method	131.928%	220.173%	6.150%	0.000%	358.25%
Multi-objective Interactive Fuzzy Programming	0.000%	0.000%	0.000%	200.000%	**200.00%**

**Fig 4:**
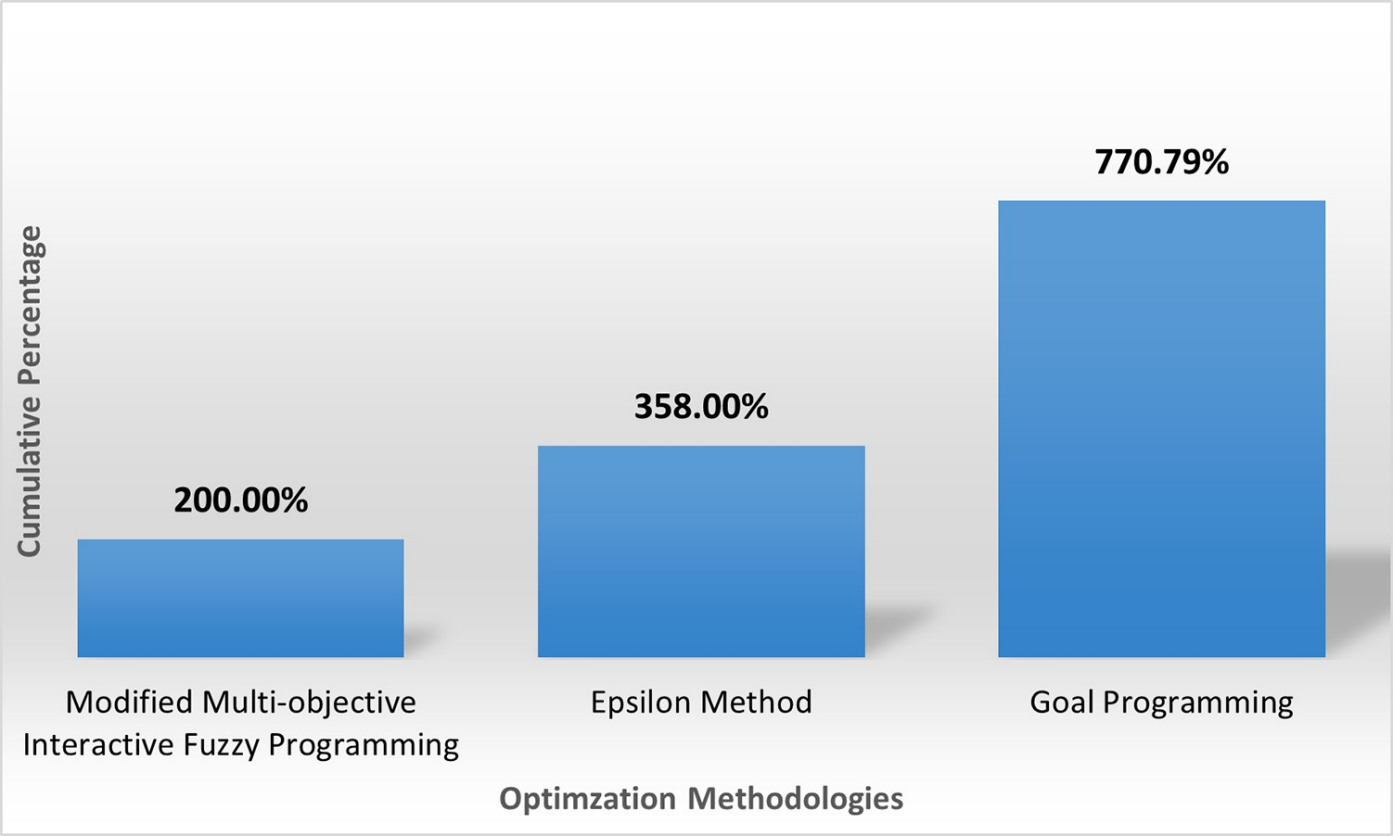
Cumulative gaps of different optimization methodologies.

Table 22 shows that the results for multi-objective interactive fuzzy weighted programming are better as compared to other methods, as the percentage gap is greater for other methodologies because the values using computed during use of other methodologies are greater. At the end, the accumulative percentage gap is found, and it is deduced that the proposed methodology is superior to other methodologies used. Fig 4 shows the plot for cumulative gaps of each methodology.

## 5. Conclusions

Supplier selection is an MCDM problem comprising of different approaches and techniques to enhance the value chain among the suppliers and customers. Green supplier selection is found to be an effective approach for reducing the environmental deterioration caused by the manufacturing industries. This study considered a textile supply chain problem for the procurement of textile dyestuff products. The availability of a good quality dyestuff product supplied by the right suppliers at the right time is the most important concern of the textile SMEs while keeping the environment safe and clean at the same time. This procurement problem evaluates a set of suppliers based on their environmental scores referred to as green appraisal scores along with the traditional economic criteria of cost, quality of the product, and manufacturing and logistics time. The quality of dyestuff products is measured by considering the quality complaints for the last year and quality inspection of the product. Moreover, this model also considered AQL which ensures the quality of the product. This business quartet of cost, quality, time, and the green score is a novel in the supply chain management literature of the textile industry. The proposed model introduced a proper environmental evaluation methodology while integrating Quality Function Deployment (QFD) with Interactive Multi-Objective Fuzzy Goal Programming Technique. The environmental parameters are evaluated based on a scale in terms of various technologies working in the world. In the proposed model, each objective function is solved individually and then set as a constraint for other objectives to obtain their extreme solutions. The Green score ranking is kept as a constraint for all objectives to select top ‘n’ suppliers with the highest green scores. The satisfaction levels are calculated using the fuzzy membership function, and weights are assigned to these satisfaction levels by calculating Aggregate Fuzzy Weights.

The model used human decision-making in the problem by incorporating their importance preferences for each objective. The model is validated on a numerical example of a GTSME (buyer) and five suppliers. The scores of all green parameters for the suppliers is computed by one-on-one interviews with the suppliers and quantified using the environmental scale. The solution of this multi-objective problem gives 100% satisfaction for cost and time whereas the satisfaction level of quality is 93.95% and for the green objective, it is 75%. The model validates the satisfaction of constraints. Changes in uncertainty is also tested in the model. Sensitivity analysis on data parameters is performed to validate the model for different values. The proposed research methodology is also compared with other optimization methods and the results are compiled. The proposed research will be helpful for decision-makers in terms of the assessment of green suppliers and demand or order allocation. This research model can be extended for optimal transportation routing strategies, inventory handling, and priority order delivery policies. The decision-makers can further include the concept of stochastic demand or distribution free demand, for example the study done by [116]. Furthermore, GSCM can be linked to Corporate Social Responsibility (CSR) factors that will give a competitive edge to the organizations.

## Supporting information

S1 Appendix (DOCX)
