# Supplementary material for: Incorporating management opinion in green supplier selection model using quality function deployment and interactive fuzzy programming
Source: PLoS One. 2022 Jun 16;17(6):e0268552. doi: 10.1371/journal.pone.0268552 (PMC9202931; doi:10.1371/journal.pone.0268552)
Supplement: S1 Appendix — (DOCX) [file pone.0268552.s001.docx]

**Appendix A**

Table A-1. The environmental scale of green parameters

| Scale for Green Parameters | | |
| --- | --- | --- |
| Green Packaging | 1 | Non-recyclable packaging |
|  | 2 | Primary packaging is recyclable |
|  | 3 | Both primary and secondary packaging is recyclable |
|  | 4 | Primary and secondary packaging is recyclable, and tertiary packaging is returnable |
| Energy & Natural Resource Consumption | 1 | No energy monitoring |
|  | 2 | Energy monitoring, Analysis, and Saving policies |
|  | 3 | Energy friendly resources used in manufacturing processes |
|  | 4 | ISO 50001 EMS Certified |
| Degree of use of environment-friendly raw materials | 1 | Non-recyclable materials |
|  | 2 | Primary Raw materials are recyclable |
|  | 3 | Primary Raw materials are Compostable or biodegradable materials |
|  | 4 | Recyclable primary raw materials using as Secondary Raw materials |
| Greenhouse Gas (GHG) emissions during handling and transportation | 1 | No GHG emissions monitoring and control |
|  | 2 | GHG emissions control policies without reduction |
|  | 3 | GHG emissions reduction policies like carbon cap and trade, carbon tax |
|  | 4 | ISO 14064 or other related certifications |
| Air Pollution Control during transportation | 1 | Air Quality Index (AQI)= 201-500: Very unhealthy to hazardous |
|  | 2 | Air Quality Index (AQI)= 101-200: Unhealthy |
|  | 3 | Air Quality Index (AQI)= 51-100: Moderate |
|  | 4 | Air Quality Index (AQI)= 0-50: Green |
| Passing ISO 14000 or other environmental Certifications | 1 | 25% target achieved |
|  | 2 | 50% target achieved |
|  | 3 | 75% target achieved |
|  | 4 | 100% target achieved |
| Degree of having environmental friendly plans & policies | 1 | 0-25% |
|  | 2 | 26-50% |
|  | 3 | 51-75% |
|  | 4 | 76-100% |
| Wastewater treatment | 1 | No water conservation, no waste water generation monitoring, and record |
|  | 2 | Water conservation objectives and policies |
|  | 3 | Recycling and reuse of natural water e.g. rainwater harvesting technologies |
|  | 4 | Wastewater treatment plant |
| Solid waste treatment | 1 | Landfills |
|  | 2 | Incineration |
|  | 3 | Composting |
|  | 4 | Recycling (resource recovery) |
